# The rising tide of frailty in Parkinson’s disease: a bibliometric study of global research landscape and emerging trends

**DOI:** 10.3389/fneur.2026.1720699

**Published:** 2026-04-09

**Authors:** Ying Jin, Rui Ren, Xijiang Tian, Dan Ye

**Affiliations:** 1 The Second Affiliated Hospital of Zhejiang Chinese Medical University, Hangzhou, China; 2 Sehan University, Yeonggam, Republic of Korea; 3 Weifang University of Science and Technology, Weifang, Shandong, China

**Keywords:** bibliometric analysis, CiteSpace, frailty, Parkinson ‘s disease, VOSviewer

## Abstract

**Background:**

Frailty represents a prevalent comorbidity in patients with Parkinson’s disease (PD) and poses an escalating global health challenge, particularly amid rapidly aging populations. Despite the increasing recognition of frailty in PD, a comprehensive bibliometric analysis of the research landscape remains absent. This study aims to systematically evaluate the current research status on frailty in PD and uncover emerging trends and focal points through bibliometric methods.

**Methods:**

On September 22, 2025, a comprehensive literature retrieval on frailty in PD, dating from January 1, 2004, was conducted using the Web of Science Core Collection (WoSCC) and Scopus databases. Bibliometric analyses were performed using CiteSpace and VOSviewer, while R software was used for additional visualization and analysis.

**Results:**

A total of 2,391 publications on frailty in PD were identified, authored by 7,379 researchers from 1,789 institutions across 72 countries. Publication output exhibited a strong upward trend over the past two decades (*R*^2^ = 0.90), with projections indicating continued growth. Early research focused on foundational biomedical and psychosocial domains, while current studies have shifted toward applied clinical and interdisciplinary areas. Key research domains include rehabilitation medicine, clinical neurology, and neuroscience. Current research hotspots center on frailty prevention, early detection, and therapeutic interventions. Additionally, emerging keywords highlight “gait,” “rehabilitation,” and the “Brain-Gut Axis” as focal points in the study of frailty in PD.

**Conclusion:**

This bibliometric analysis provides a comprehensive overview of research progress in PD-related frailty, highlighting key themes and future directions. The field increasingly focuses on early diagnosis, rehabilitation, and mechanistic studies, particularly the Brain-Gut Axis’s role in frailty development. These insights offer valuable insights to guide future research, promote interdisciplinary collaboration, and inform strategies for improving patient care and disease management.

## 1 Introduction

As the global population continues to age, frailty has emerged as a substantial global health challenge, carrying significant implications for both clinical practice and public health (1). Frailty is a condition characterized by increased vulnerability due to the decline of multiple physiological systems, which impairs an individual’s ability to maintain internal balance in response to stressors (1). It is commonly considered a transitional state between independence and severe disability, and is closely associated with various risk factors such as multimorbidity, low socioeconomic status, poor nutrition, and a sedentary lifestyle. Frailty shares overlapping symptoms with Parkinson’s disease (PD), including both motor and non-motor disorders, and is one of the most prevalent comorbidities in patients with PD, with incidence rates ranging from 29 to 67% (2–5). Additionally, the relationship between frailty and PD is complex, with studies indicating a positive correlation between frailty and both the risk and severity of PD (6–8). It is anticipated that the prevalence of frailty among patients with PD will rise sharply in the coming decades, in parallel with the increasing incidence of PD (9, 10). Evidence suggests that factors such as motor impairment, cognitive dysfunction, polypharmacy, adverse drug reactions, low physical activity, fatigue, slow gait speed, and reduced grip strength are associated with a higher prevalence of frailty in patients with PD (6, 7, 11). Frailty not only profoundly impacts the quality of life of patients with PD by elevating the risks of falls, disability, hospitalization, and premature death, but also intensifies the demand for care, thus increasing the healthcare burden and complicating the clinical management of patients with PD (3, 12, 13). Consequently, the prevention and delay of frailty in patients with PD is critical for improving prognosis and quality of life.

Recent research has explored strategies to prevent and mitigate frailty in patients with PD, with existing evidence suggesting that early interventions, such as physical activity and nutritional support, may serve as potential strategies for frailty prevention and management. However, the underlying mechanisms of frailty in patients with PD remain unclear, and effective preventive measures are still lacking. Current studies are fragmented across different disciplines, with limited integration of neurological, nutritional, and geriatric perspectives, highlighting the need for a systematic overview of research trends and knowledge gaps in this field (1, 13). Over the past two decades, there has been a significant increase in research focused on frailty in patients with PD. However, no comprehensive and integrated analyses of key authors, institutions, research progress, and future development trends within this field are still lacking, leaving research directions and focal points unclear. Therefore, a bibliometric analysis is crucial to achieving a comprehensive understanding of the current state of research, identifying hotspots, and clarifying future research trajectories in the study of frailty in patients with PD.

Bibliometrics is a well-established methodology for both qualitative and quantitative analysis of scientific literature. It offers a systematic and comprehensive assessment of an entire research field, aiding in the identification of development trends and the prediction of future directions (14). This approach has been extensively applied across various medical disciplines, such as medical education, dentistry, medical imaging, digital health technologies, and sleep disorder, to evaluate research status and guide future studies (15–19). Previous bibliometric analyses have highlighted scientific advancements in several PD-related areas, including accidental falls, acupuncture therapy, surgical treatments, ferroptosis, speech disorders, and dysphagia, focusing on temporal trends, geographic distribution, and contributing factors (20–25). These studies, however, differ from this study in several methodological aspects, such as data sources and study periods. However, to date, no bibliometric study has examined the knowledge structure, evolution pathways, and research hotspots specifically related to frailty in patients with PD. Over the past two decades, significant progress has been made in understanding frailty in this context, yet a comprehensive analysis is still lacking. Therefore, this study seeks to systematically analyze the research on frailty in patients with PD from the past 20 years using bibliometric methods. Constructing a knowledge map of the field will uncover development trends and predict project research directions, offering researchers novel insights and perspectives for further exploration of frailty in PD.

## 2 Materials and methods

### 2.1 Data collection

**Figure 1:**
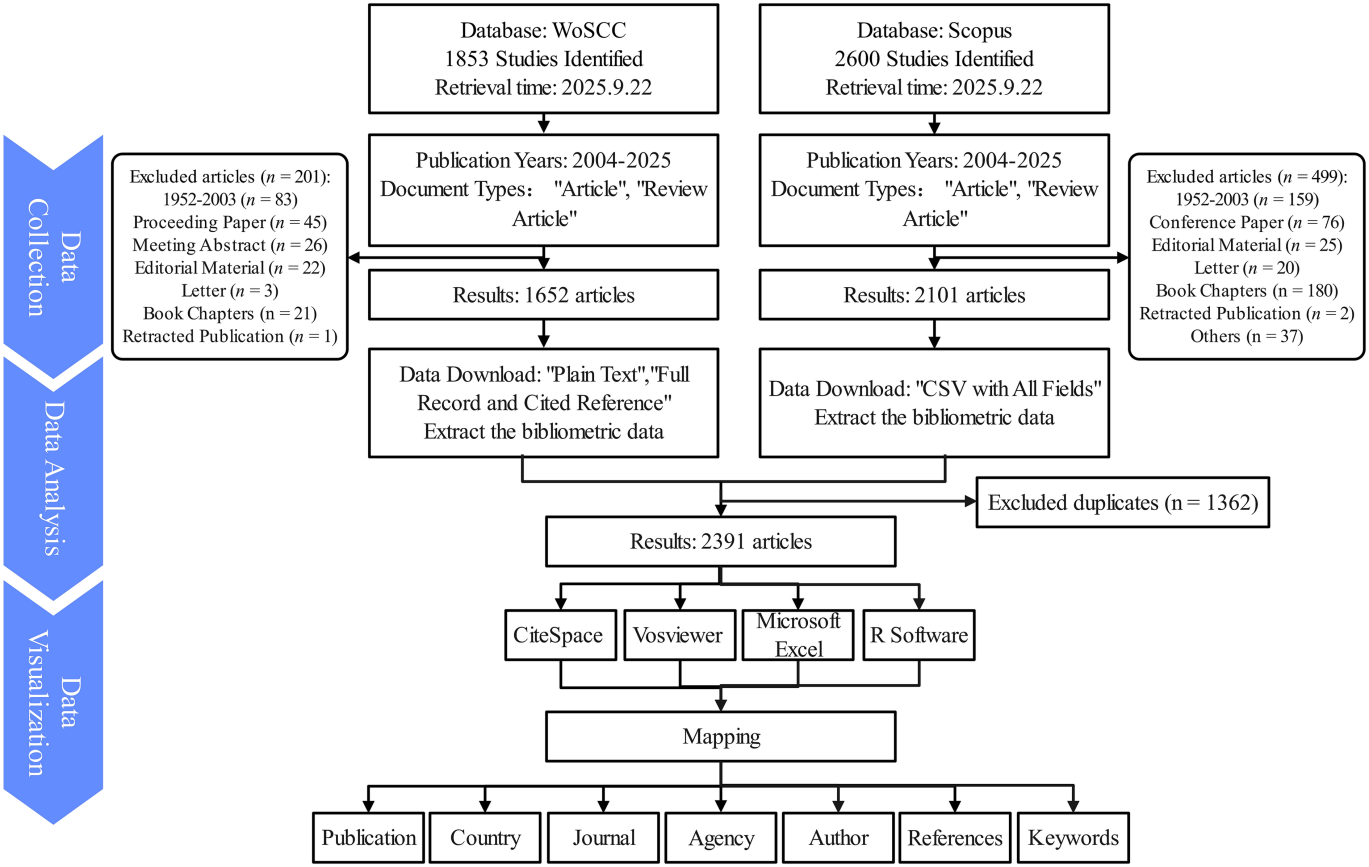
Inclusion and exclusion processes of research on frailty in patients with Parkinson’s disease. The flowchart illustrates the data source, inclusion and exclusion criteria, final dataset selection, and key phases of bibliometric analysis, including co-authorship, keyword clustering, and co-citation analysis. This figure visually summarizes the methodological framework and analytical workflow used in the study.

After identifying the title keywords from the relevant articles, MESH subject terms from PubMed were utilized to refine and validate the search strategy. Literature searches and data collection were conducted through the Web of Science Core Collection (WoSCC) and Scopus database, two of the most extensive and comprehensive scientific literature databases globally. On September 22, 2025, a detailed search was carried out in WoSCC using the following strategy: (((TI = (“Frailt*” OR “Debilit*” OR “Astheni*” OR “Lassitud*” OR “Frailty Index” OR “Physical frailty”)) OR AK = (“Frailt*” OR “Debilit*” OR “Astheni*” OR “Lassitud*” OR “Frailty Index” OR “Physical frailty”)) OR AB = (“Frailt*” OR “Debilit*” OR “Astheni*” OR “Lassitud*” OR “Frailty Index” OR “Physical frailty”)) AND TS = (Parkinson*). In Scopus, the search was performed using “Article title, Abstract, Keywords.” The initial WoSCC search retrieved 1,853 records. After applying filters for document type (articles and reviews), excluding retracted publications, and restricting the time span to 2004–2025, 1,652 articles were retained for analysis (Figure 1). The Scopus search was performed using the following query string: TITLE-ABS-KEY (Parkinson*) AND TITLE-ABS-KEY (“Frailt*” OR “Debilit*” OR “Astheni*” OR “Lassitud*” OR “Frailty Index” OR “Physical frailty”). Document types were limited to articles and reviews, excluding retracted publications, and restricting the time span to 2004–2025. In Scopus, a total of 2,600 publications were identified, and after apply the same filters, 2,101 publications remained.

All selected literature was exported from WoSCC in both “Plain Text” and “Full Record and Cited Reference” formats, while Scopus records were exported as “CSV with All Fields.” Duplicate entries were removed using EndNote 2025 based on DOI, title, and first author, resulting in a final dataset of 2,391 publications for bibliometric analysis. In addition, author names and institutional affiliations were standardized to minimize inconsistencies due to variations in spelling or abbreviations across databases. Key bibliometric metrics, including the number of publications, citation counts, and H-index, were subsequently extracted. The literature search and data extraction processes were independently conducted by two researchers, with any discrepancies reviewed by a third party for resolution and consensus among all three participants.

### 2.2 Data analysis

CiteSpace 6.3. R1, VOSviewer 1.6.20, Microsoft Excel 2019 and R Software were employed to conduct bibliometric and knowledge mapping analyses. Prior to keyword analysis, synonyms were merged, and variations in author and institution spellings were standardized. Keyword synonyms were harmonized using a VOSviewer thesaurus file, and inconsistencies in author and institutional names were corrected through manual verification combined with software-assisted normalization to ensure consistency across databases. To ensure methodological consistency, the same time span (2000–2025) was applied across all bibliometric analyses conducted using CiteSpace, VOSviewer, and Microsoft Excel. Due to software compatibility constraints, CiteSpace analyses (including co-citation networks and burst detection) were conducted using WoSCC data only, whereas VOSviewer analyses (co-authorship, keyword co-occurrence, and collaboration networks) were based on the merged dataset from WoSCC and Scopus. Despite differences in data sources, the key patterns observed were consistent across analyses, providing confidence in the robustness of our bibliometric findings. All figures and tables explicitly indicate whether the results were derived from WoSCC-only data or from the combined WoSCC–Scopus dataset.

#### 2.2.1 Microsoft Excel 2019

Microsoft Excel 2019 was used to assess publication volume and citation frequency and to model and predict future publication trends through curve fitting.

#### 2.2.2 CiteSpace

CiteSpace facilitated the examination of the distribution, contributions, and collaborations among countries, institutions, authors, and categories, while also providing centrality, burst citation, timeline, cluster dependency, and hotspot analysis of keywords and co-cited references (26). The strength metric was applied to indicate the frequency of keyword occurrences, while the temporal distribution of keywords was captured by the start and end times. Hotspots were identified by high-frequency keywords in key scientific fields.

#### 2.2.3 VOSviewer

VOSviewer, developed by Nees Jan van Eck and Ludo Waltman at Leiden University, served as an additional tool for bibliometric analysis. It constructs and visualizes bibliometric networks based on data, performing keyword cluster analysis based on their occurrences in titles and abstracts (27). Keyword frequency and relationships were visually represented by the size, color, and connections of the circles. This software also enabled the analysis of collaborative clusters among authors and institutions.

#### 2.2.4 Software tools and parameter settings

To improve methodological clarity and reproducibility, the key parameters for each software tool are summarized below. CiteSpace 6.3. R1 was used for co-citation analysis, citation burst detection, and keyword hotspot analysis. Parameters included the time slice was set from 2004 to 2025, with one-year intervals (#Years Per Slice = 1). Node types encompassed authors, countries/regions, institutions, and keywords, while selection criteria were configured as Top *N* = 50 with g-index: *k* = 25. Pruning techniques, including “Pathfinder” and “Prune sliced networks,” were employed, generating visual maps upon execution. Parameter settings in CiteSpace and VOSviewer were determined based on prior bibliometric studies and preliminary trials to ensure network stability and interpretability (26, 27).

## 3 Results

### 3.1 Annual publication growth trend

**Figure 2:**
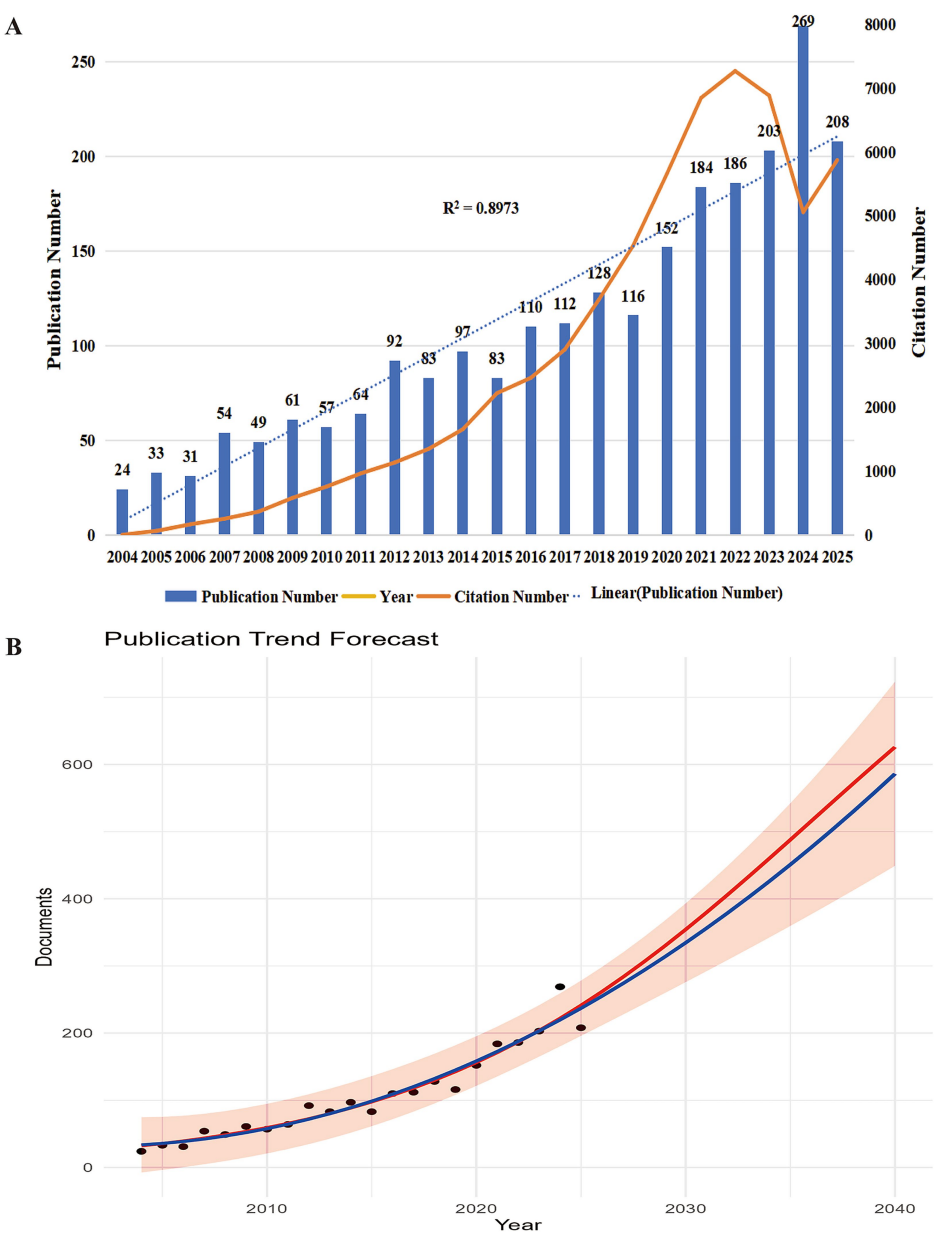
Temporal trends and future projection of research output related to frailty in patients with Parkinson’s disease. **(A)** Annual number of publications (bars) and citations (line) from 2005 to 2025, highlighting steady growth in scholarly attention to this field. **(B)** Trend prediction of annual publications on frailty in patients with Parkinson’s disease (2026–2040). Black dots represent actual publication counts from 2005 to 2025, while the red curve illustrates the projected annual publication trend. The prediction indicates a steady increase, suggesting sustained and expanding research interest in this field.

The annual trend of research output offers a straightforward yet insightful overview of global activity and scientific focus within a specific research field (14). Analyzing publication volume and citation counts enables the tracking of a domain’s developmental trajectory. In this study, 2,391 articles related to frailty in PD were retrieved from the WoSCC and Scopus databases, boasting an H-index of 108, a total of 54,794 citations, and an average citation count of 38.16 per article. Figure 2A illustrates the annual number of publications and citations between 2004 and September 22, 2025. The correlation coefficient (R^2^) of publication volume over this period is 0.90, indicating a strong upward trend. Notably, the number of publications in 2024 (269) was more than 10 times greater than in 2004 (24). Citation counts have also steadily increased annually, peaking in 2022, reflecting sustained research interest in frailty among patients with PD. Figure 2B shows a descriptive extension of historical publication trends using a linear model. This visualization is intended to contextualize past growth patterns rather than to provide a definitive forecast of future research output.

### 3.2 Analysis of countries

**Figure 3:**
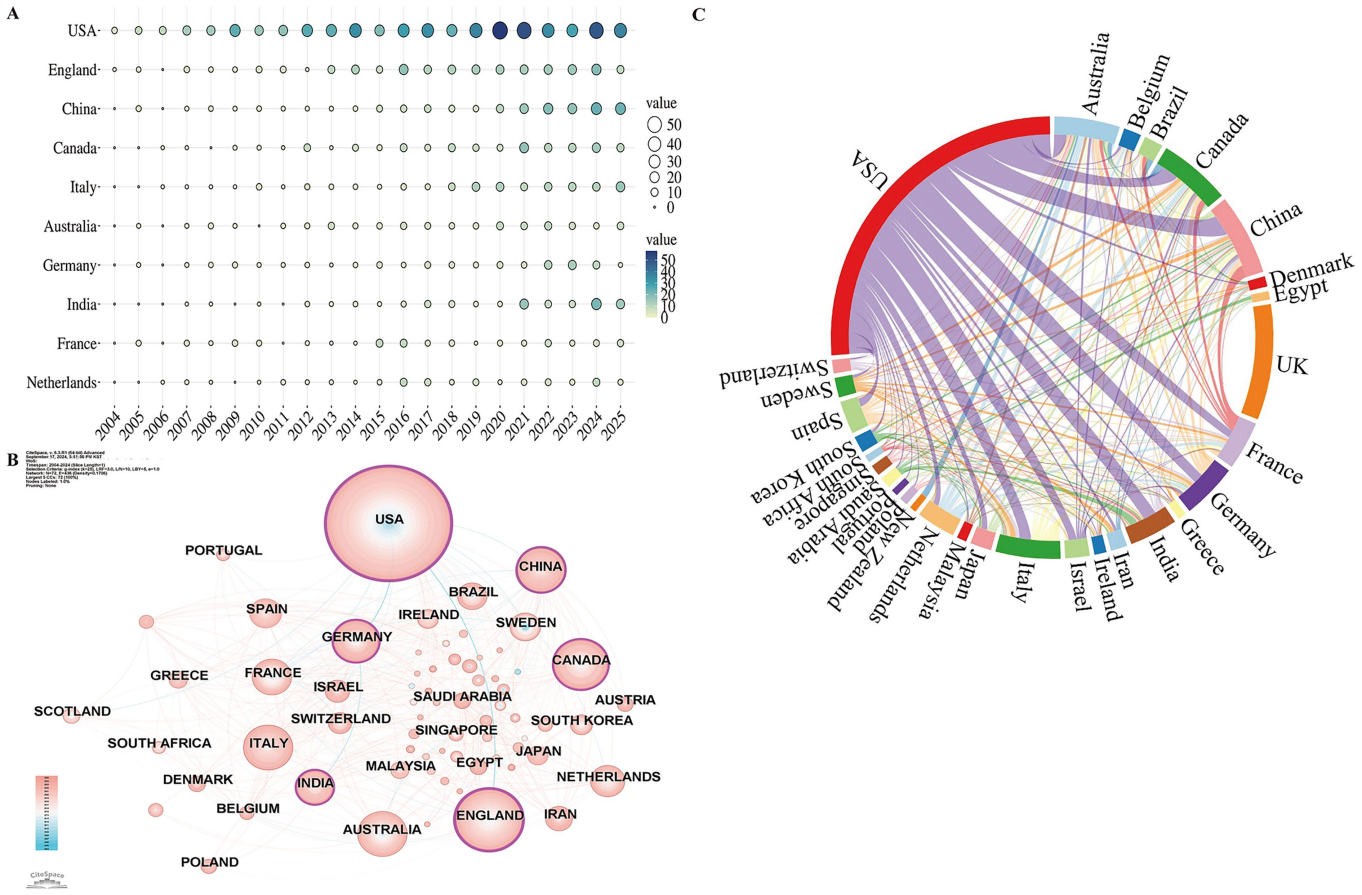
Geospatial analysis of research output on frailty in Parkinson’s disease. **(A)** Temporal trends in annual publication output from the top 10 most prolific countries. Node size reflects the annual publication count. **(B)** Co-occurrence network of contributing countries. Node size corresponds to the frequency of co-occurrence, while nodes encircled in purple indicate high centrality (≥0.1), signifying influential bridging roles. **(C)** Collaboration network among countries, where link thickness indicates the strength of collaboration. Colors represent collaboration clusters, with major clusters corresponding to the main international research partnerships.

The geographic distribution of research highlights the most influential countries and sheds light on trends in international collaboration. A total of 72 countries have contributed to this research area. Figure 3A presents the annual publication volume for the top 10 countries, all of which exhibit a steady rise in output. The United States, the United Kingdom, and China consistently lead in publication volume. According to Supplementary Table S1, the United States (542 publications) ranks first, producing three times as many papers as United Kingdom (179), which holds second place. China follows in third with 133 publications. In terms of citation impact, the United States (25,643 citations) leads by a wide margin, followed by United Kingdom (10,872) and Italy (6,123). The H-index rankings mirror these trends, with the United States (86) and United Kingdom (50) occupying the top two positions, and Italy (36) ranking third.

Nodes with relatively high betweenness centrality occupy structurally important positions within the constructed network under the specified parameter settings, indicating their potential role as connectors among research themes. Figure 3B visualizes the publication volume and centrality of each country, where the node size corresponds to publication volume and purple nodes indicate centrality scores ≥ 0.1. Six countries demonstrate high centrality: the United States (0.52), United Kingdom (0.27), India (0.16), Canada (0.11), Germany (0.10), and China (0.10), underscoring their pivotal roles in frailty research in PD. Figure 3C illustrates international collaborations, with the strongest partnerships observed between the United States, United Kingdom and China, confirming that the United States and United Kingdom are at the forefront of research in this field.

### 3.3 Analysis of institutions

The analysis of institutional distribution highlights the leading research institutions, offering valuable insights for potential collaborations. A total of 1,789 institutions have contributed to this research field. Supplementary Table S2 lists the top 10 institutions in terms of publication volume, citation counts, and H-index. The University College London leads in publication volume with 49 papers, followed by the University of Toronto (45) and the Centre National de la Recherche Scientifique (CNRS) (41). Regarding citation counts, the University of Cambridge ranks first with 5,019 citations, while the University College London (2,568) and CNRS (2,493) occupy the second and third positions, respectively. In terms of the H-index, the University College London (26) and CNRS (24) are again the leaders, with Harvard University (23) ranking third.

**Figure 4:**
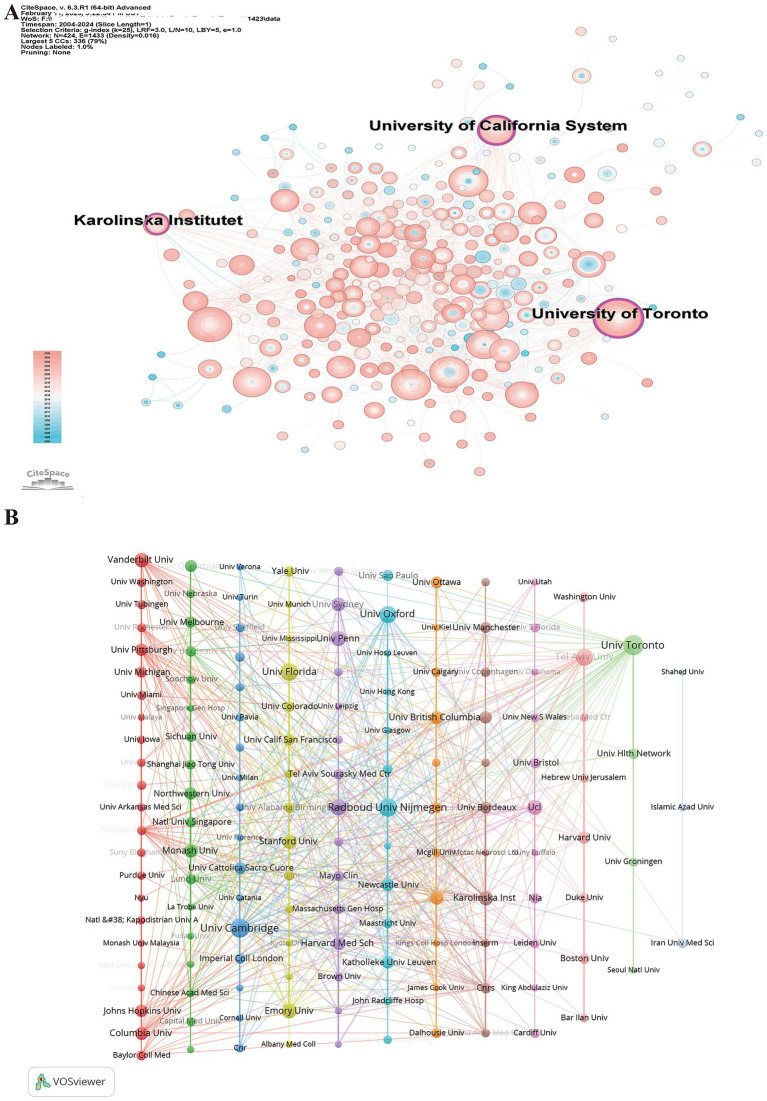
Institutional collaboration network in research on frailty in Parkinson’s disease. **(A)** Co-occurrence network of contributing institutions. Node size represents the frequency of co-occurrence, with purple-encircled nodes indicating high centrality (≥0.1), demonstrating influential bridging roles. **(B)** Co-occurrence network of contributing institutions, with node colors representing different institutional clusters based on collaborative patterns. Node size reflects the total publication output, while link thickness indicates the strength of collaboration between institutions. Colors represent clusters identified using the VOSviewer clustering algorithm based on collaboration strength.

Figure 4A illustrates the publication volume and centrality of each institution. Notably, three institutions have a centrality score of ≥0.1: the University of Toronto (0.20), the University of California System (0.15), and the Karolinska Institutet (0.10), underscoring their pivotal roles in frailty research in patients with PD. Figure 4B depicts the collaboration network among 160 institutions with an occurrence frequency of ≥4. Different colors represent distinct clusters, node sizes correspond to publication volume, and the thickness of the connecting lines reflects the strength of co-occurrence relationships between institutions. These institutions are organized into 12 clusters based on the strength of their collaborations. The largest cluster (red), consisting of 23 institutions, is led by Johns Hopkins University and Vanderbilt University. The second largest cluster (green), with 18 institutions, is anchored by Monash University and Northwestern University, while the third largest cluster (blue), comprising 17 institutions, is headed by the University of Cambridge.

### 3.4 Analysis of journals

**Figure 5:**
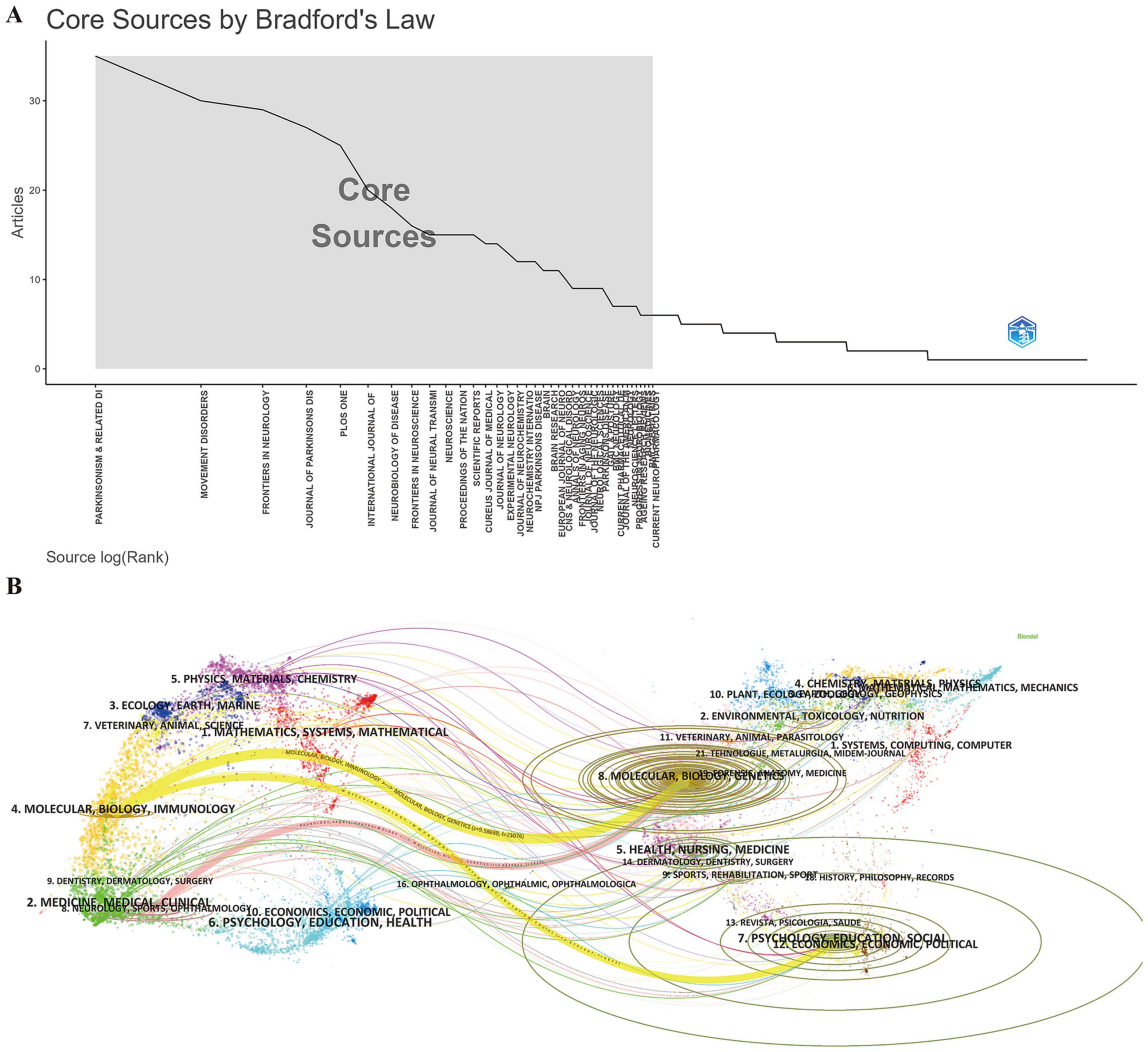
Academic journals related to frailty in Parkinson’s disease. **(A)** Bradford’s law according to the academic journals. **(B)** Dual-map overlay of the citation landscape in frailty research within Parkinson’s disease (WoSCC-only). On the left, clusters represent citing journal groups, while on the right, clusters represent the most frequently cited journals. Colored lines connecting the two maps illustrate the citation relationships between citing and cited journal clusters.

The visualization of published journals and co-cited journals aids in identifying the most active and influential publications in the field of frailty among patients with PD. Figure 5A and Supplementary Table S3 lists the top 10 journals contributing to PD frailty research. *Parkinsonism & Related Disorders* leads with 34 publications, followed by *Movement Disorders* (30) and *Frontiers in Neurology* (29). In terms of Journal Citation Reports (JCR) Quartile rankings, five journals are classified as Q1, and five as Q2. *Movement Disorders* holds the highest citation count (1,606), followed by *Parkinsonism & Related Disorders* (1,123) and *Neurobiology of Disease* (1,097). The highest H-index belongs to *Parkinsonism & Related Disorders* (20), with *Movement Disorders* (19) and *Neurobiology of Disease* (14) in second and third, respectively.

Given the database integration constraints, the dual-map overlay of journals was generated using WoSCC data (Figure 5B). The map shows citing journals on the left and cited journals on the right, with three colored paths depicting citation flows. The cited fields mainly include Molecular, Biology, Genetics/Psychology, Education, Social/Molecular, Biology, Genetics, forming the knowledge foundation for PD frailty research. The citing fields, including Molecular, Biology, Immunology/Neurology, Sports, Ophthalmology, point to the current research frontiers in this area. This pattern indicates a disciplinary shift in PD frailty research, with knowledge increasingly transferring from foundational biomedical and psychosocial domains toward applied clinical and interdisciplinary areas.

### 3.5 Analysis of authors

Author publication activity and collaboration analysis provides insight into the most influential contributors, aiding in the identification of key experts. A total of 7,379 authors have contributed to research in this field. Supplementary Table S4 lists the top 10 authors by publication volume and citation count, with Erwan Bezard (France) and Bastiaan R. Bloem (Netherlands) ranking highly in both metrics, indicating their prominence in the field.

**Figure 6:**
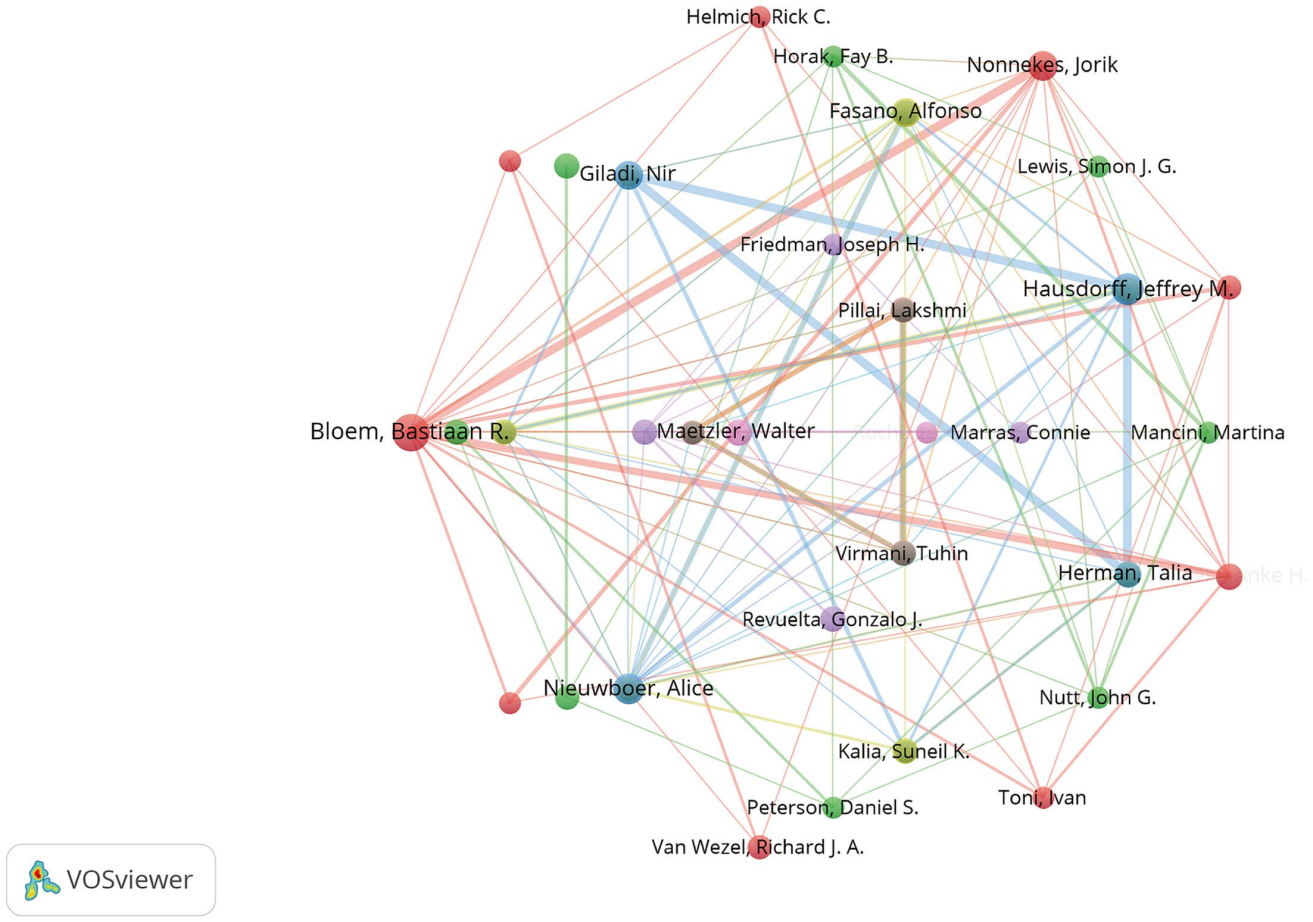
Co-authorship network of contributing authors in research on frailty in Parkinson’s disease. Nodes are colored by cluster membership, reflecting collaborative patterns. Node size corresponds to the frequency of co-authorship, while links indicate relationships between authors based on co-authored publications.

Figure 6 depicts the collaboration network of 194 authors with at least three publications, organized into 9 clusters. The largest cluster (red) includes 9 authors, with Bloem, Bastiaan R and Nonnekes, Jorik factor at its core. The second-largest cluster (green), led by Earhart, Gammon M and Hassin-Baer, Sharon, consists of 8 authors and shows strong collaboration with the cluster centered on Bastiaan R. Bloem. The third-largest cluster (blue) comprises 7 authors, led by Hausdorff, Jeffrey M and Nieuwboer, Alice, who also maintains close collaborations with other key clusters.

### 3.6 Analysis of reference

For researchers in this field, highly cited articles hold considerable importance. Supplementary Table S5 lists the top 10 most-cited papers, with the top two articles receiving over 1,000 citations each. Of these, six are research articles, and four are reviews (28–37). These influential studies can be grouped into six major categories: molecular mechanisms of neurodegeneration, experimental PD models, systemic regulatory pathways, aging-related processes, clinical intervention strategies, and methodological tools. Key contributions include Knowles et al. (36), Chiti and Dobson (37), which clarified the role of protein misfolding and amyloid aggregates in neurodegenerative disorders, and Haynes et al. (32), which highlighted cellular mechanisms protecting against oxidative stress. Cannon et al. (33) provided a reproducible rotenone PD model, while Martin et al. (34) emphasized the emerging role of the brain–gut–microbiome axis in frailty. Franceschi et al. (31) discussed aging and age-related disease mechanisms, and Bauman et al. (29) together with Cummings et al. (30) offered insights into interventions promoting functional maintenance. Finally, methodological advances such as those by Aguado et al. (28) and Conchillo-Sole et al. (35) underpin experimental and computational approaches in the field. These studies have advanced mechanistic understanding, informed experimental designs, and guided potential intervention strategies, collectively shaping the research landscape and development trajectory of frailty in PD.

**Figure 7:**
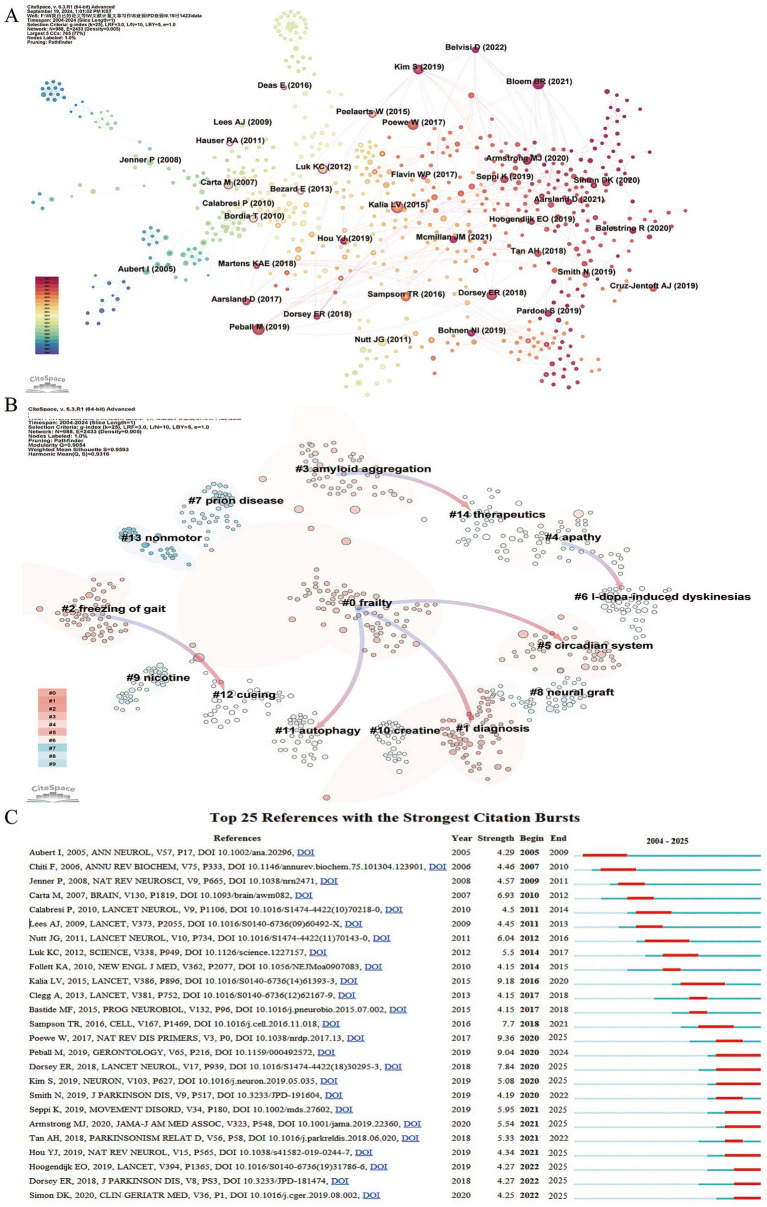
Reference analysis in frailty research in Parkinson’s disease. **(A)** Co-citation network visualization (WoSCC-only). Node size reflects citation frequency, with purple-encircled nodes indicating high centrality (≥0.1), signifying influential works bridging different research clusters. Node color transitions from pink to blue represent temporal patterns, with pink marking more recent publications. **(B)** Clustering of references based on semantic similarity. Pink arrows denote the primary citation flow within each cluster (WoSCC-only). **(C)** Top 25 references with the strongest citation bursts (WoSCC-only). Red bars highlight periods of significantly elevated citation counts, revealing emergent publications that garnered intense scholarly attention over a specific timeframe.

To further explore the intellectual structure of the field, a co-citation network was generated based on the WoSCC database (Figure 7A). Fifteen papers demonstrate strong centrality, with the most central being “Pathological α-synuclein transmission initiates Parkinson-like neurodegeneration in nontransgenic mice,” authored by Luk KC and published in *Science* in 2012. This paper, with a centrality of 0.40, established a mechanistic link between the transmission of pathological α-synuclein and key PD features, emphasizing the neurodegenerative process (38).

Co-citation cluster analysis offers an objective view of the knowledge structure within this field (14). Based on interrelationships between papers, 15 clusters were identified, with arrows indicating dependencies among them (Figure 7B). Cluster #0 (frailty) emerged from Cluster #1 (diagnosis), Cluster #5 (circadian system), and Cluster #11 (autophagy). Cluster #2 (freezing of gait) evolved from Cluster #12 (cueing), while Cluster #4 (apathy) developed from Cluster #6 (l-dopa-induced dyskinesia). However, Clusters #0 (frailty), #2 (freezing of gait), and #4 (apathy) did not further evolve, indicating they represent current research frontiers in the field.

Citation bursts highlight references that have experienced a sharp increase in citations over a certain period, shown as red bars that mark the peak years of citation bursts (14). In the “Top 25 References with the Strongest Citation Bursts” (Figure 7C), the earliest burst occurred in 2005. The strongest burst (9.36) was from the 2017 article “Parkinson disease” by Werner Poewe, published in *Nature Reviews Disease Primers*. This paper emphasized α-synuclein aggregation and cellular transport as therapeutic targets, identifying markers for the prodromal phase of the disease and enabling earlier intervention with disease-modifying therapies (39). The longest burst, lasting 4 years (2020–2024), was from the 2018 article by Marina Peball, published in *Gerontology,* titled “Prevalence and Associated Factors of Sarcopenia and Frailty in Parkinson’s Disease: a Cross-Sectional Study” (5). Currently, nine papers are still experiencing citation bursts, including eight reviews and one research article. Notable examples include David K. Simon’s 2020 article in *Clinics in Geriatric Medicine*, titled “Parkinson Disease Epidemiology, Pathology, Genetics, and Pathophysiology” (40), and Emiel O. Hoogendijk’s 2019 article in *The Lancet*, titled “Frailty: implications for clinical practice and public health” (1). These works collectively highlight current research hotspots, focusing on the epidemiology and pathological mechanisms of frailty in patients with PD, the relationship between frailty and non-motor symptoms of PD, and the role of aging, indicating significant areas of ongoing investigation.

### 3.7 Analysis of hotspots and frontiers

**Figure 8:**
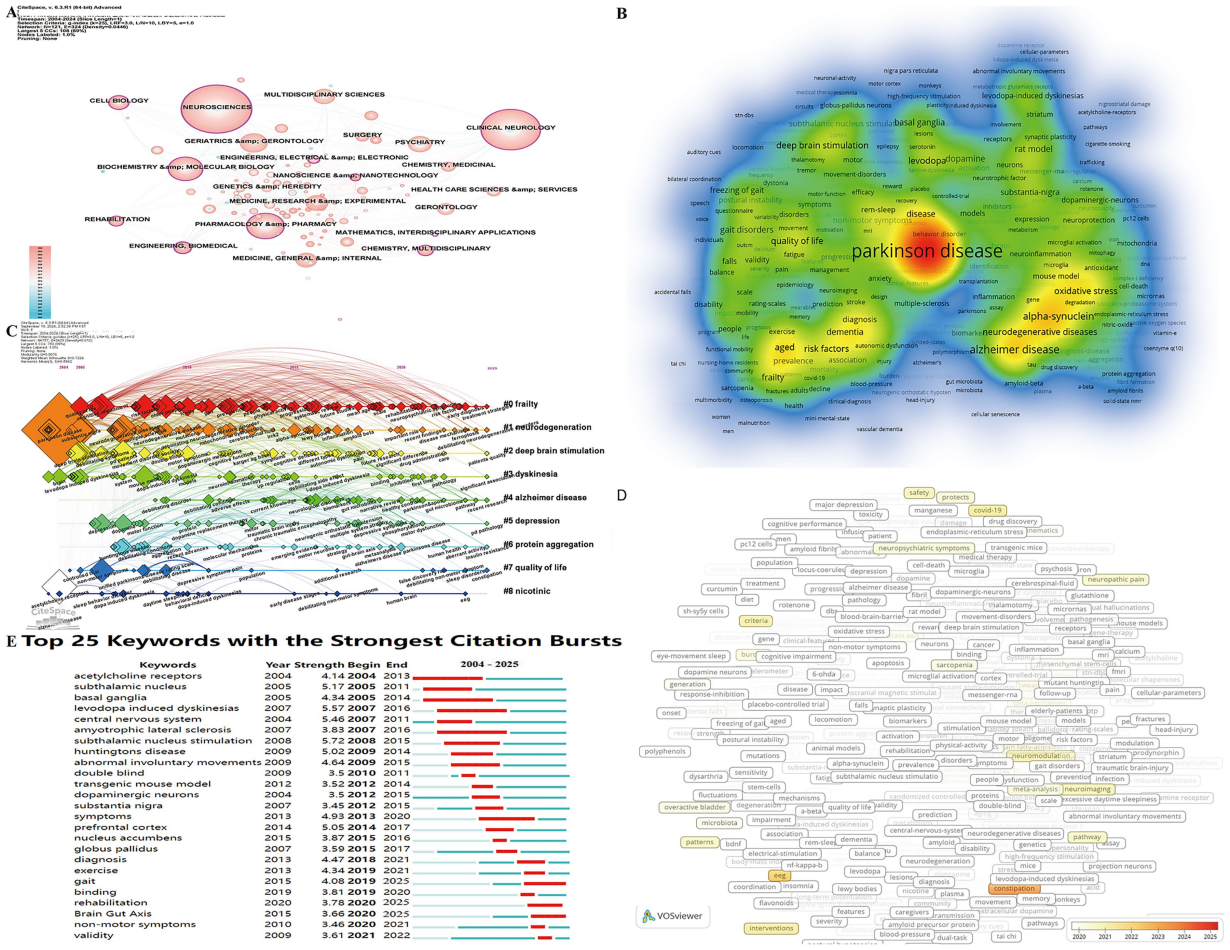
Analytical overview of research domains and key terms in the study of frailty in Parkinson’s disease. **(A)** Distribution of research fields and disciplines (WoSCC-only). Node size represents citation frequency, while purple nodes signify high centrality (≥0.1), highlighting influential works bridging different research clusters. **(B)** Co-occurrence density map of prominent keywords in the field. Keywords with higher occurrence frequencies are shown in warmer colors (yellow/red), while lower-frequency keywords appear in cooler colors (green/blue). **(C)** Temporal view showing the emergence and evolution of key terms over time. **(D)** Overlay visualization showing temporal evolution of keywords. **(E)** Top 25 keywords with the strongest citation bursts. Red bars indicate periods of significantly elevated citation counts for specific keywords, signaling emerging research trends that attracted considerable academic attention.

Currently, 129 research categories are engaged in the study of frailty in patients with PD (Figure 8A). Among these, 11 categories exhibit strong centrality, with the top six fields being Neurosciences (0.34), Engineering, Biomedical (0.23), Biochemistry & Molecular Biology (0.18), Pharmacology & Pharmacy (0.16), Rehabilitation (0.16), and Clinical Neurology (0.15). These fields represent the core areas of focus in current research, highlighting the interdisciplinary nature of frailty research in PD.

Keywords provide insight into the themes and content of research articles and analyzing their co-occurrence can reveal research hotspots and trends (14). A keyword density map (Figure 8B) was created based on keyword frequency. Excluding “Parkinson disease” and “frailty,” the top 10 keywords were Alzheimer’s disease (263) substantia nigra (146) deep brain stimulation (145) cognitive impairment (145) oxidative stress (138) risk factors (126) dopaminergic neurons (123) non-motor symptoms (122) mouse model (112) and alpha-synuclein (108). These keywords primarily reflect the pathogenesis pathological features clinical manifestations and therapeutic interventions for frailty in patients with PD indicating these are the current research hotspots and frontiers.

The keyword timeline viewer further helps analyze the temporal evolution of research clusters and tracks the progression of key research focuses at different stages (14). Figure 8C shows that eight of the nine clusters remain active, with the exception of Cluster #8 (nicotinic). The largest cluster, Cluster #0 (frailty), initially focused on keywords like “quality of life” and “cognitive impairment,” but recent research has shifted toward “risk factors,” “early diagnosis,” and “treatment strategies.” Clusters #3 (dyskinesia) and #1 (neurodegeneration) represent some of the earliest and most cited research areas, with early key keywords such as “substantia nigra,” “brain,” “levodopa-induced dyskinesias,” and “mouse model.” Cluster #4 (Alzheimer’s disease) is the most recent, with prominent keywords like “gut microbiome” and “pathway.” The progression of research keywords illustrates a clear shift from earlier studies focused on risk factors, pathological mechanisms, and clinical manifestations to a current emphasis on early prevention and therapeutic interventions for frailty in patients with PD.

VOSviewer was employed to visualize keyword trends in frailty research in patients with PD from 2020 to 2025, based on the Average Appearance Year (AAY), with keywords marked by color (Figure 8D). Keywords shown in white appeared earlier, while red indicate more recent terms. Notable terms gaining popularity in recent years include “sarcopenia,” “machine learning,” “freezing of gait,” “kinematics,” “generation,” “constipation,” “pathway,” “mitophagy,” and “wearable sensors.” These terms are primarily associated with frailty prevention (e.g., sarcopenia, mitophagy), early diagnosis (e.g., machine learning, constipation, kinematics), and treatment strategies (e.g., generation, pathway, wearable sensors). This pattern aligns with the emergence of keywords like “risk factor,” “early diagnosis,” and “treatment strategies” in the largest cluster (#0 frailty) depicted in the keyword timeline map.

Another key indicator of emerging research hotspots is the presence of keywords with strong citation bursts (14). CiteSpace was utilized to extract citation bursts for all keywords, with a focus on the top 25 (Figure 8E). The earliest burst began in 2004 with “acetylcholine receptors.” Notably, “levodopa-induced dyskinesias” (2007–2016) and “basal ganglia” (2005–2014) exhibited the longest-lasting bursts, while “subthalamic nucleus stimulation” had the strongest burst (5.72). Current citation bursts include terms like “gait,” “rehabilitation,” and “Brain-Gut Axis,” indicating these are critical research hotspots and trends. By analyzing the evolution of keyword bursts over time, the development of this research field can be divided into three stages. The early exploratory stage (2004–2010) predominantly focused on neurophysiological mechanisms, particularly basal ganglia circuitry, acetylcholine receptors, and dopaminergic neuron degeneration. As the field progressed, during the development stage (2011–2017), research attention shifted toward pathological features and clinical manifestations, including levodopa-induced movement disorders and the relationship between Parkinsonian motor and non-motor symptoms with frailty. In the recent expansion stage (2018–2025), growing emphasis on early prevention and therapeutic interventions, such as the influence of gait abnormalities on frailty, the effectiveness of rehabilitation in mitigating frailty symptoms, and the role of the brain-gut axis in the frailty of patients with PD.

## 4 Discussion

### 4.1 Research of frailty in patients with PD is currently garnering significant attention and interests

In recent years, the number of publications on frailty among patients with PD has grown significantly, with a notable global surge since 2015. The fitted trend line suggests that this momentum will likely continue, driven in large part by the global aging population, where frailty is increasingly recognized as a critical indicator of PD progression (13).

Globally, multiple countries have launched research initiatives in this field, with the United States and the United Kingdom taking leading roles. In terms of institutional centrality, the University of Toronto in Canada and the University of California System in the United States exhibit high intermediary centrality, indicating their dominant positions in the research landscape. Notably, four of the top 10 most prolific authors are based in the United States, while Erwan Bezard from France and Bastiaan R. Bloem from the Netherlands have emerged as key figures in this domain. Beyond differences in productivity and collaboration patterns, research priorities may also vary across countries and institutions. For instance, studies from North America and Western Europe often emphasize mechanistic investigations and clinical characterization of frailty in PD, whereas research from other regions increasingly focuses on rehabilitation strategies, functional assessment, and clinical management. These variations may reflect differences in healthcare systems, research funding priorities, and population health needs. Consequently, their ongoing contributions will likely continue to shape the field’s future, making these institutions and scholars prime candidates for collaboration.

Researchers should also pay close attention to certain high-impact journals in this field. Core publications such as *Parkinsonism Related Disorders*, *Movement Disorders*, and *Neurobiology of Disease* (listed in Supplementary Table S3 and Figure 5A) are central to the study of frailty in PD, with a focus on clinical practice, rehabilitation, and immunology. These journals align with findings from the dual journal overlay analysis (Figure 5B), which suggests a current focus on basic research, though clinical applications are increasingly becoming prominent. Therefore, these journals are recommended for submissions, and following recent publications in them is essential for staying updated on emerging trends.

As highlighted in Supplementary Table S5, the top 10 most-cited references primarily explore the pathological mechanisms and potential interventions for frailty in patients with PD, including four review articles. Among them, Luk et al.’s (38) *Science* paper, “Pathological α-synuclein transmission initiates Parkinson-like neurodegeneration in nontransgenic mice,” has the highest centrality (0.40), establishing a pivotal connection between pathological α-synuclein propagation and the core features of PD, forming a cornerstone for subsequent research (37). Analysis of citation bursts reveals that references in this field are frequently cited over extended periods, indicating sustained scientific interest and dynamic shifts in frailty research among patients with PD. The earliest citation burst began in 2007, while a 2018 article experienced a burst lasting 4 years. Currently, nine references are still undergoing citation bursts, eight of which are reviews. These works provide comprehensive insights into frailty’s epidemiology, pathological mechanisms, and its relationship with non-motor symptoms and aging in patients with PD, pointing to ongoing research hotspots and key areas of interest.

### 4.2 Hotspots and trends

Bibliometrics plays a vital role in quantitatively and systematically analyzing research trends within specific fields by integrating bibliometric data with visualization techniques, providing valuable insights into potential future research directions. Currently, studies on frailty in PD are primarily concentrated in fields such as Neurosciences, Biomedical Engineering, Biochemistry & Molecular Biology, Pharmacology & Pharmacy, Rehabilitation, and Clinical Neurology (Figure 8A). One key aspect of this approach is the analysis of frequently occurring keywords, which reveals evolving trends and dominant themes within a scholarly field. To gain a comprehensive understanding of how research on frailty in patients with PD has progressed from 2004 to 2025, it is essential to first examine the broad evolution of keywords. As depicted in Figure 7C, the initial stages of frailty research in PD centered around neurophysiological mechanisms, particularly the basal ganglia circuits, acetylcholine receptors, and dopaminergic neuron degeneration. Over time, attention shifted to exploring the pathological characteristics and clinical manifestations of frailty, including its relationship with levodopa-induced motor disorders, Parkinsonian motor and non-motor symptoms, and frailty itself. More recently, research has focused on the prevention of frailty in patients with PD (e.g., sarcopenia, mitophagy), early diagnosis (e.g., machine learning, constipation, kinematics), and treatment strategies (e.g., generation, pathway, wearable sensors), as shown in Figures 8C,D. In the past 5 years, research on topics such as “gait,” “rehabilitation,” and the “brain-gut axis” has gained significant attention (Figure 8E). Key research terms revolve around rehabilitation medicine, clinical neurology, and neuroscience, indicating the interdisciplinary nature of frailty research in PD and its growing clinical and translational relevance. From a mechanistic perspective, these research hotspots represent complementary dimensions of frailty development in PD: the gut–brain axis reflects potential biological pathways linking neurodegeneration and systemic vulnerability, gait impairment represents an integrated functional manifestation of motor and non-motor decline associated with frailty, and rehabilitation strategies focus on enhancing physiological resilience and functional capacity. Together, these emerging directions suggest a shift in PD frailty research from descriptive characterization toward a more integrated framework combining biological mechanisms, functional assessment, and intervention-oriented strategies.

#### 4.2.1 The role of the gut-brain axis in frailty among patients with PD

PD is a neurodegenerative disorder marked by both motor and non-motor symptoms, including gastrointestinal complications. In recent years, the gut-brain axis has gained considerable attention due to its pivotal role in the pathogenesis and progression of PD (41). As illustrated in Figure 8E, “Brain-Gut Axis” is currently a keyword experiencing a citation burst, while Figure 8C highlights “gut microbiome” and “pathway” as emerging terms within the cluster focused on Alzheimer’s disease (#4), underscoring an increasing research interest in this area. These patterns indicate an emerging research emphasis on gut-related mechanisms in neurodegenerative and aging-related conditions, rather than direct evidence of causality.

Existing experimental and clinical studies have explored how the gut environment may influence central nervous system function through microbiome-associated physiological processes, gut barrier regulation, and peripheral neuronal signaling (42–44). Within this context, frailty has been increasingly discussed as a condition that may intersect with PD through alterations in gut function and systemic vulnerability (6). Notably, recent publications have examined dietary modulation, microbiome-related interventions, and molecular regulatory pathways as potential areas of investigation in PD-associated frailty (45). Studies exploring probiotics, prebiotics, and nutritional supplementation—including alpha-ketoglutarate and curcumin—have contributed to this growing body of literature (46–50). However, the prominence of these topics in the literature reflects research interest and hypothesis generation rather than confirmed therapeutic benefit, which cannot be assessed through bibliometric analysis alone. Despite this progress, research on the relationship between gut dysbiosis and frailty in patients with PD remains in its early stages (51). Future studies investigating the underlying molecular mechanisms will be essential for advancing treatment efficacy and improving early diagnostic capabilities. Moreover, exploring non-invasive preventive interventions represents an underexplored but potentially critical area in the management of frailty in patients with PD, offering avenues for improving patient outcomes. The observed trends highlight an evolving research trajectory rather than established intervention efficacy.

#### 4.2.2 The impact of gait disorder on frailty in patients with PD

Our findings suggest that “gait” has gained increasing academic attention in PD research since 2015 and remains a key focus area (Figures 8D,E). In the co-citation cluster analysis, Cluster #2 (freezing of gait) has not evolved into other clusters (Figure 7B), underscoring its significance as a current research hotspot. Gait abnormalities are among the most prevalent motor disorders in patients with PD, severely impacting walking ability, increasing fall risk, and exacerbating frailty symptoms (1, 5, 52, 53). These gait disorders are influenced by a variety of factors, including the patient’s age at onset, motor symptoms, cognitive function, depression, anxiety, social functioning, and psychological factors (54–57). Therefore, gait analysis plays a crucial role in the detection and severity prediction of PD (53). Consequently, gait analysis is critical for detecting PD and predicting disease severity. Recent research has explored various methods to assess and address gait dysfunction in PD. Emerging technologies such as gait analysis systems and wearable devices have shown promise in evaluating gait abnormalities and related frailty symptoms (58–62). The growing prominence of these terms reflects an expanding methodological and technological focus within PD research, rather than validated improvements in frailty outcomes. Interventions designed to treat and improve gait abnormalities in frail patients with PD including pharmacological treatments, exercise regimens, gait training, virtual reality, neuromodulation, observational learning, movement imagery, and technology-assisted cognitive-motor dual-task (CMDT) rehabilitation, as well as deep brain stimulation have been frequently discussed in the literature (63–70). While these approaches are often framed as potentially beneficial for mobility and quality of life, their appearance in bibliometric networks should be interpreted as indicators of research activity rather than evidence of effectiveness in alleviating frailty. However, limited research has focused specifically on frailty symptoms in different subgroups of patients with PD (71). Future research should prioritize understanding the underlying mechanisms of gait abnormalities and incorporate comprehensive gait analysis to develop targeted interventions. Such approaches could enhance physical function and alleviate frailty symptoms in patients with PD, ultimately improving their overall quality of life.

#### 4.2.3 Rehabilitation in preventing frailty in patients with PD

Rehabilitation has increasingly become a vital element in the comprehensive management of PD, encompassing various therapies such as exercise, physical, occupational, and speech therapy (72). Our study highlights that “treatment strategies” has emerged as a prominent keyword in the largest cluster (#0, frailty) (Figure 8C), while rehabilitation is currently experiencing a citation burst (Figure 8E), underscoring its importance as a research focus.

Insufficient physical activity is frequently discussed in the literature as a contributing factor to frailty progression in older adults and in individuals with PD (73, 74). Consequently, exercise-based interventions have been widely investigated in PD research, with studies examining traditional modalities such as dance, yoga, strength training, and tai chi, as well as newer technology-assisted approaches (75–77). Emerging rehabilitation strategies, including exergaming, virtual reality-assisted exercise, tablet-based home rehabilitation programs, and combined physical-cognitive training, have been increasingly reported in recent studies (78–80). In addition, speech and psychological interventions have been discussed in relation to communication difficulties and cognitive decline in PD (81). The growing diversity of rehabilitation-related keywords reflects an expanding and multidisciplinary research landscape. Factors influencing adherence to rehabilitation, such as visual feedback, psychological resilience, and social support, have also gained attention in recent reviews and empirical studies (79, 82). Accessibility and utilization of rehabilitation services are also essential for patients with PD (83). However, no single approach can fully address all symptoms, and research on combining various interventions to effectively treat frailty remains limited (76, 84). Future studies should emphasize multidisciplinary collaboration, integrating innovative technologies, psychological interventions, and personalized rehabilitation plans. This approach can enhance both physical and cognitive function, improve patient participation and autonomy, and ultimately elevate the quality of life for patients with PD.

#### 4.2.4 Bibliometric inference limitations

It should be acknowledged that bibliometric analysis is primarily designed to map knowledge structures, research emphasis, and thematic evolution based on published literature. Although approaches such as keyword burst detection and co-citation analysis are effective in identifying emerging topics and intellectual trends, they do not allow causal inference or evaluation of clinical effectiveness. Consequently, topics highlighted in this study, including the gut-brain axis, nutritional interventions, gait-related research, and rehabilitation should be interpreted as indicators of scholarly attention and evolving research priorities, rather than as evidence supporting specific biological mechanisms or therapeutic benefits. Finally, bibliometric analysis primarily reflects the quantitative characteristics and structural patterns of the literature and does not include a detailed evaluation of the methodological quality or level of evidence of individual studies. Future studies may integrate bibliometric approaches with systematic reviews or meta-analyses to provide a more comprehensive understanding of the research field.

## 5 Research limitations and future prospects

In this study, bibliometric analysis and visualization were combined to offer a systematic overview of frailty symptoms in patients with PD, enabling readers to grasp research progress and emerging trends with ease. Additionally, the study includes a stratified analysis of different regions, institutions, journals, and researchers, providing valuable insights into potential collaboration opportunities and reliable data for scientists. However, several limitations should be acknowledged. First, given the open nature of databases, our analysis does not account for new publications released after the retrieval date, thus reflecting only the current state of research at the time of data collection. Second, this study primarily used the merged dataset from WoSCC and Scopus to improve coverage and reliability, some analyses could only be performed using WoSCC data because of software limitations. WoSCC-only analyses may have influenced the identification of influential references and the structure of citation networks to a limited extent. Nevertheless, the major descriptive analyses in this study were based on the combined dataset, which helps reduce potential database-related bias. Lastly, this study did not perform a systematic analysis of funding agency distributions across the field. Future bibliometric studies may explore funding patterns to further understand how research investment shapes the development of frailty research in PD.

Despite these limitations, several directions for future research can be proposed. First, integrating multiple bibliographic databases with interoperable analytical tools may improve coverage and robustness. Second, more targeted mechanistic investigations are needed to address key research gaps in emerging hotspots. For example, studies on the gut-brain axis should further explore how gut microbiota composition, microbial metabolites, and neuroinflammatory pathways contribute to frailty progression in patients with PD. In addition, although rehabilitation interventions have shown promise in improving mobility and functional capacity, further research is required to determine optimal intervention intensity, individualized rehabilitation protocols, and strategies to enhance long-term adherence in clinical settings. Finally, longitudinal, multi-center studies and clinical trials are needed to validate these potential mechanisms and intervention strategies, thereby facilitating the clinical translation of frailty research in PD.

Another critical challenge in this field is the fragmentation of existing research, which arises from differences in study designs, assessment tools for frailty, and disciplinary perspectives. Future studies should prioritize the development of standardized frailty assessment frameworks specifically tailored for patients with Parkinson’s disease. In addition, interdisciplinary collaboration among neurologists, geriatricians, rehabilitation specialists, and microbiome researchers may help integrate currently dispersed research themes. Establishing large-scale longitudinal cohorts and shared data platforms could further facilitate the synthesis of evidence and promote a more coherent research framework for frailty in PD.

## 6 Conclusion

In summary, this bibliometric study highlights the research hotspots and trends in the field of frailty among patients with PD over the past two decades. The findings show that research in this area is experiencing rapid global growth and is poised to continue its positive trajectory in the near future. The United States and the United Kingdom are at the forefront of this field, with institutions such as the University of Toronto and the University of California System playing key roles in advancing frailty research in patients with PD. Currently, rehabilitation medicine, clinical neurology, and neuroscience represent the major research areas. As the field evolves, there is an increasing emphasis on the prevention, early diagnosis, and therapeutic interventions for frailty in patients with PD. This study provides an objective and comprehensive overview of the current research landscape, offering scholars valuable insights and guidance for future research in this rapidly advancing field.

## Data Availability

The raw data supporting the conclusions of this article will be made available by the authors, without undue reservation.
