# Supplementary material for: The rising tide of frailty in Parkinson’s disease: a bibliometric study of global research landscape and emerging trends
Source: Front Neurol. 2026 Apr 9;17:1720699. doi: 10.3389/fneur.2026.1720699 (PMC13102578; doi:10.3389/fneur.2026.1720699)
Supplement: Supplementary file 1 [file Table_1.docx]

| **Rank** | **Countries** | **Np** | **Countries** | **Nc** | **Countries** | **H-Index** | **Countries** | **Total Link Strength** |
| --- | --- | --- | --- | --- | --- | --- | --- | --- |
| 1 | USA | 542 | USA | 25,643 | USA | 86 | USA | 302 |
| 2 | England | 179 | England | 10872 | England | 50 | England | 236 |
| 3 | China | 133 | Italy | 6123 | Italy | 36 | Canada | 149 |
| 4 | Canada | 113 | Canada | 3973 | China | 34 | Germany | 140 |
| 5 | Italy | 102 | France | 3622 | Canada | 33 | France | 133 |
| 6 | Australia | 93 | China | 3534 | Germany | 31 | Italy | 122 |
| 7 | Germany | 86 | Spain | 3144 | Australia | 30 | China | 112 |
| 8 | India | 79 | Australia | 3080 | France | 30 | Netherlands | 92 |
| 9 | France | 74 | Germany | 2946 | Spain | 27 | Spain | 84 |
| 10 | Netherlands | 64 | Netherlands | 2812 | Netherlands | 26 | Australia | 75 |

**Supplementary Table S1. The Top10 countries in the research of fralty in patients with Parkinson Disease**
