# Supplementary material for: The rising tide of frailty in Parkinson’s disease: a bibliometric study of global research landscape and emerging trends
Source: Front Neurol. 2026 Apr 9;17:1720699. doi: 10.3389/fneur.2026.1720699 (PMC13102578; doi:10.3389/fneur.2026.1720699)
Supplement: Supplementary file 2 [file Table_2.docx]

**Supplementary Table S2. The Top10 Institutions in the research of fralty in patients with Parkinson Disease**

| **Rank** | **Institution** | **Np** | **Country** | **Institution** | **Nc** | **Institution** | **H-Index** |
| --- | --- | --- | --- | --- | --- | --- | --- |
| 1 | University College London | 49 | England | University Of Cambridge | 5019 | University College London | 26 |
| 2 | University Of Toronto | 45 | Canada | University College London | 2568 | Centre National De La Recherche Scientifique Cnrs | 24 |
| 3 | Centre National De La Recherche Scientifique Cnrs | 41 | France | Centre National De La Recherche Scientifique Cnrs | 2493 | Harvard University | 23 |
| 4 | State University System Of Florida | 39 | USA | Harvard University | 2191 | University Of Cambridge | 23 |
| 5 | University Of California System | 38 | USA | University Of California System | 1984 | University Of Toronto | 20 |
| 6 | Harvard University | 36 | USA | University Of Toronto | 1938 | US Department Of Veterans Affairs | 20 |
| 7 | US Department Of Veterans Affairs | 35 | USA | Radboud University Nijmegen | 1846 | University Of California System | 19 |
| 8 | Radboud University Nijmegen | 32 | Netherlands | US Department Of Veterans Affairs | 1589 | State University System Of Florida | 18 |
| 9 | University Of Cambridge | 32 | England | University Of Oxford | 1260 | Radboud University Nijmegen | 18 |
| 10 | University Of Oxford | 29 | USA | State University System Of Florida | 1241 | University Of Oxford | 16 |
