# Supplementary material for: The rising tide of frailty in Parkinson’s disease: a bibliometric study of global research landscape and emerging trends
Source: Front Neurol. 2026 Apr 9;17:1720699. doi: 10.3389/fneur.2026.1720699 (PMC13102578; doi:10.3389/fneur.2026.1720699)
Supplement: Supplementary file 3 [file Table_3.docx]

**Supplementary Table S3. The Top10 Journals in the research of fralty in patients with Parkinson Disease**

| **Rank** | **Journal** | **Np** | **%of1243** | IF（JCR2023) | **JCR Quatile** | **Journal** | **Nc** | **Journal** | **H-Index** |
| --- | --- | --- | --- | --- | --- | --- | --- | --- | --- |
| 1 | Parkinsonism Related Disorders | 34 | 2.57% | 3.1 | Q2 | Movement Disorders | 1606 | Parkinsonism Related Disorders | 20 |
| 2 | Movement Disorders | 30 | 2.33% | 7.4 | Q1 | Parkinsonism Related Disorders | 1123 | Movement Disorders | 19 |
| 3 | Frontiers In Neurology | 29 | 2.17% | 2.7 | Q2 | Neurobiology Of Disease | 1097 | Neurobiology Of Disease | 14 |
| 4 | Journal Of Parkinsons Disease | 27 | 2.01% | 4.0 | Q2 | Brain | 1003 | Plos One | 12 |
| 5 | Plos One | 25 | 1.93% | 2.9 | Q1 | Journal Of Neurochemistry | 908 | Frontiers In Neurology | 12 |
| 6 | International Journal Of Molecular Sciences | 20 | 1.37% | 4.9 | Q1 | Plos One | 785 | International Journal Of Molecular Sciences | 12 |
| 7 | Neurobiology Of Disease | 18 | 1.37% | 5.1 | Q1 | Neuroscience | 637 | Journal Of Parkinsons Disease | 11 |
| 8 | Frontiers In Neuroscience | 16 | 1.21% | 3.2 | Q2 | International Journal Of Molecular Sciences | 589 | Neuroscience | 11 |
| 9 | Scientific Reports | 15 | 1.13% | 3.8 | Q1 | Frontiers In Neuroscience | 545 | Journal Of Neurology | 11 |
| 10 | Journal Of Neural Transmission | 15 | 1.05% | 3.2 | Q2 | Journal Of Parkinsons Disease | 462 | Scientific Reports | 10 |
