# Supplementary material for: The rising tide of frailty in Parkinson’s disease: a bibliometric study of global research landscape and emerging trends
Source: Front Neurol. 2026 Apr 9;17:1720699. doi: 10.3389/fneur.2026.1720699 (PMC13102578; doi:10.3389/fneur.2026.1720699)
Supplement: Supplementary file 4 [file Table_4.docx]

**Supplementary Table S4. The Top10 Authors in the research of fralty in patients with Parkinson Disease**

| **Rank** | **Author** | **Np** | **Country** | **Author** | **H-Index** | **Country** | **Author** | **times cited** | **Country** |
| --- | --- | --- | --- | --- | --- | --- | --- | --- | --- |
| 1 | Bezard, Erwan | 22 | France | Bezard, Erwan | 15 | France | Dobson, Christopher M | 4124 | England |
| 2 | Bloem, Bastiaan R | 17 | Netherlands | Bloem, Bastiaan R | 13 | Netherlands | Michele Vendruscolo | 2177 | England |
| 3 | Quik, Maryka | 13 | USA | Quik, Maryka | 13 | USA | Bezard, Erwan | 1750 | France |
| 4 | Hausdorff, Jeffrey | 12 | USA | Dobson, Christopher M | 10 | England | Quik, Maryka | 1450 | USA |
| 5 | Dobson, Christopher M | 11 | England | Hausdorff, Jeffrey | 9 | England | Bloem, Bastiaan R | 1311 | Nijmegen |
| 6 | Nieuwboer, Alice M | 10 | Belgium | Nieuwboer, Alice M | 8 | Belgium | Bennett, David A | 1000 | USA |
| 7 | Okun, Michael S | 10 | USA | Fasano, Alfonso | 8 | Canada | Aron S Buchman | 927 | USA |
| 8 | Rana, Abdul | 8 | Canada | Bennett, David A | 8 | USA | Guigoni, Celine | 766 | France |
| 9 | Fasano, Alfonso | 8 | Canada | Okun, Michael S | 7 | USA | Hausdorff, Jeffrey | 739 | USA |
| 10 | Bennett, David A | 8 | USA | Nonnekes, Jorik | 7 | Netherlands | Fasano, Alfonso | 655 | Canada |
