# Supplementary material for: The rising tide of frailty in Parkinson’s disease: a bibliometric study of global research landscape and emerging trends
Source: Front Neurol. 2026 Apr 9;17:1720699. doi: 10.3389/fneur.2026.1720699 (PMC13102578; doi:10.3389/fneur.2026.1720699)
Supplement: Supplementary file 5 [file Table_5.docx]

**Supplementary Table S5. The Top10 Articles in the research of fralty in patients with Parkinson Disease**

| **Rank** | **Title** | **First Author** | **Journal** | **Year** | **Nc** | **Document Type** | **DOI** |
| --- | --- | --- | --- | --- | --- | --- | --- |
| 1 | Protein Misfolding, Amyloid Formation, and Human Disease: A Summary of Progress Over the Last Decade | Chiti, Fabrizio | Annual Review Of Biochemistry | 2017 | 1745 | Review | 10.1146/annurev-biochem-061516-045115 |
| 2 | The amyloid state and its association with protein misfolding diseases | Knowles, Tuomas P. J | Nature Reviews Molecular Cell Biology | 2014 | 1731 | Review | 10.1038/nrm3810 |
| 3 | AGGRESCAN:: a server for the prediction and evaluation of hot spots of aggregation in polypeptides | Conchillo-Sole, Oscar | Bmc Bioinformatics | 2007 | 776 | Article | 10.1186/1471-2105-8-65 |
| 4 | The Brain-Gut-Microbiome Axis | Martin, Clair R. | Cellular And Molecular Gastroenterology And Hepatology | 2018 | 698 | Review | 10.1016/j.jcmgh.2018.04.003 |
| 5 | A highly reproducible rotenone model of Parkinson's disease | Cannon, Jason R. | Neurobiology Of Disease | 2009 | 554 | Article | 10.1016/j.nbd.2009.01.016 |
| 6 | Improving Viability of Stem Cells During Syringe Needle Flow Through the Design of Hydrogel Cell Carriers | Aguado, Brian A | Tissue Engineering Part A | 2012 | 539 | Article | 10.1089/ten.tea.2011.0391 |
| 7 | Degradation of misfolded proteins prevents ER-derived oxidative stress and cell death | Haynes, CM | Molecular Cell | 2004 | 524 | Article | 10.1016/j.molcel.2004.08.025 |
| 8 | The Continuum of Aging and Age-Related Diseases: Common Mechanisms but Different Rates | Franceschi, Claudio | Frontiers In Medicine | 2018 | 515 | Review | 10.3389/fmed.2018.00061 |
| 9 | Pimavanserin for patients with Parkinson's disease psychosis: a randomised, placebo-controlled phase 3 trial | Cummings, Jeffrey | Lancet | 2014 | 457 | Article | 10.1016/S0140-6736(13)62106-6 |
| 10 | Updating the Evidence for Physical Activity: Summative Reviews of the Epidemiological Evidence, Prevalence, and Interventions to Promote Active Aging | Bauman, Adrian | Gerontologist | 2016 | 455 | Article | 10.1093/geront/gnw031 |
